# Electronic exoneuron based on liquid metal for the quantitative sensing of the augmented somatosensory system

**DOI:** 10.1038/s41378-023-00535-x

**Published:** 2023-09-15

**Authors:** Jin Shang, Lixue Tang, Kaiqi Guo, Shuaijian Yang, Jinhao Cheng, Jiabin Dou, Rong Yang, Mingming Zhang, Xingyu Jiang

**Affiliations:** 1 Shenzhen Key Laboratory of Smart Healthcare Engineering, Guangdong Provincial Key Laboratory of Advanced Biomaterials, Department of Biomedical Engineering, Southern University of Science and Technology, No. 1088, Xueyuan Rd., Nanshan District, Shenzhen, Guangdong 518055 P. R. China; 2 CAS Center for Excellence in Nanoscience, Center of Materials Science and Optoelectronics Engineering, National Center for Nanoscience and Technology, University of Chinese Academy of Sciences, Beijing, 100190 P. R. China; 3 Sino-Danish Center for Education and Research, Sino-Danish College, University of Chinese Academy of Sciences, Beijing, 100190 P. R. China; 4 School of Biomedical Engineering, Capital Medical University, No.10 Xitoutiao, You An Men Wai, Beijing, 100069 China; 5 School of Biomedical Sciences, Faculty of Biological Sciences, University of Leeds, Leeds, LS2 9JT UK

**Keywords:** Electrical and electronic engineering, Electronic properties and materials

## Abstract

The increasing demands in augmented somatosensory have promoted quantitative sensing to be an emerging need for athletic training/performance evaluation and physical rehabilitation. Neurons for the somatosensory system in the human body can capture the information of movements in time but only qualitatively. This work presents an electronic Exo-neuron (EEN) that can spread throughout the limbs for realizing augmented somatosensory by recording both muscular activity and joint motion quantitatively without site constraints or drift instability, even in strenuous activities. Simply based on low-cost liquid metal and clinically used adhesive elastomer, the EEN could be easily fabricated in large areas for limbs. It is thin (~120 μm), soft, stretchable (>500%), and conformal and further shows wide applications in sports, rehabilitation, health care, and entertainment.

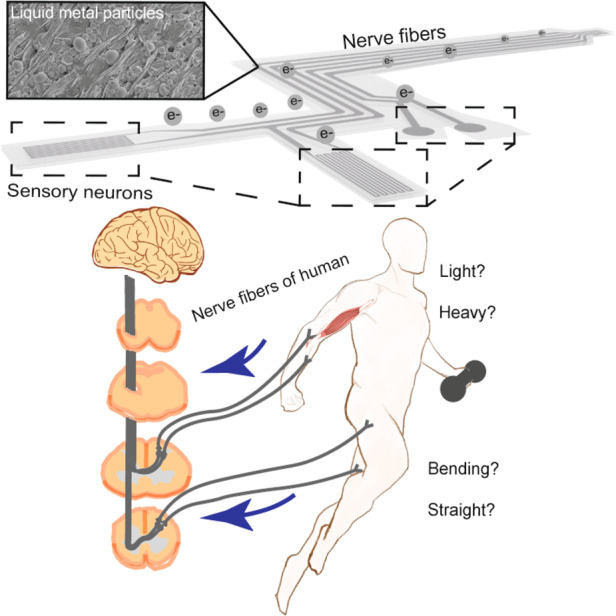

## Introduction

The somatosensory system in the human body facilitates the perception of different sensory modalities, including external stimuli (outside of the body), internal stimuli (inside of the body), and proprioception (body position and balance). As one of the basic remarkable abilities of humans, proprioception is the perception of movements of the human body, such as muscular activity and joint motion^1^. Analyzing proprioception facilitated by the somatosensory system has already been regarded as a method for evaluation^2^ in disease diagnosis, physical rehabilitation, sports training, and entertainment. However, human somatosensory neurons can only facilitate the qualitative perception of movements, which limits the objectivity and accuracy of evaluation and further cognition.

Meeting the increasing need for quantified information and digitalized analysis of human proprioception could result in enormous developmental potential in many fields. In sports, the results of racewalking athletes are generally judged according to the subjective observation of referees^3^, who are often questioned due to subjectivity. Thus, a device for an objective appraisal is strongly needed. In the field of medicine, motion sensing that incorporates both joint motion and muscular activity can provide critical information on strokes, Parkinson’s disease, and many other neuromuscular diseases^4^. Even in the field of entertainment, quantified motion sensing can further revolutionize the form of games^5^ and improve the special effects in films with fantasy scenes.

Many external devices have been developed to enhance the somatosensory system and to enable the ability of humans to quantify movements^6–10^. Some novel artificial neurons^11–13^ were developed to mimic real human neurons and were shown to enhance both external stimuli and internal stimuli but could not quantify proprioception. An optical motion capture system^14^ was developed that could track activities through markers placed on the human body (the gold standard for motion tracking) with costly cameras equipped in a specific site or fixed in laboratories. Wearable inertial measurement units^15,16^ (IMUs) were developed that could eliminate the site constraint but suffered from extensive data processing for filtering, an integrated drift caused by motion-associated artifacts, and electromagnetic interference. New soft electronics methods have also been developed^8,17–20^, such as those employing carbon nanomaterials, silver, gold, copper, hydrogels, and polymer conductors. These methods enabled artificial sensing and stimuli and further provided valuable information on human activities. However, simultaneously realizing certain properties, such as conformality, stretchability, repeatability, and breathability, is still a challenge in this field. Complex synthesis and fabrication methods also increase the costs and threshold of commercial use in real-life applications.

Here, we report an electronic exoneuron (EEN) for the quantitative sensing of the augmented somatosensory system. Spreading throughout the skin of the body, it realized the quantification of the proprioception of the human somatosensory system in a very simple way. The simple use of a clinically used thin elastomeric substrate as the epineurium of the EEN allowed this device to be safe and conform to the epidermis with great repeatability and stretchability, avoiding errors due to detachment. Gallium-based liquid metals (metals in a liquid state at room temperature) have shown excellent advantages when applied in stretchable conductors^21–27^ with great biocompatibility and conductivity. They have been successfully utilized in a wide range of applications^28–32^ both in vivo and in vitro. In this work, we chose a gallium-based liquid alloy to form a stretchable metal–polymer conductor (MPC). In the EEN, the MPC was wrapped in the epineurium to form vimineous nerve fibers for signal transmission connecting with sensory neurons related to joint motion and muscular activity. The sensory neurons were made of MPC-based motion capture modules and electrodes. Thus, the whole EEN device was similar to a neural network distributed in a specific perception area throughout the skin. It could be both easily and quickly fabricated by large-scale screen printing and large-area hot-pressing within a total of 15 min. Furthermore, it was highly commercialize and had a low cost of approximately 0.8 dollars. The outstanding features of the EEN, compared to other soft electronics-based devices, were that it could cover a large area, reach a maximum stretch of 500% of its original length and accommodate repeated application (>500 times). Targeting both joint motion and muscular activity, it could stably measure the movement of lower limbs and accurately sense both physical and electrophysiological signals in daily life for a long period of ~12 h and even for strenuous racewalking athletes. It overcame the drawbacks of optical motion capture systems (such as restriction to defined sites equipped with cameras and high cost). In addition, based on the principle of sensing the deformation of the skin outside the joints, it also showed more stable, accurate sensing properties without integral drift and further data filtering than traditional wearable devices, such as IMUs, which totally depend on the calculation of accelerometers. In this way, the EEN provides a novel tool for augmented somatosensory proprioception sensing.

## Results

**Fig. 1:**
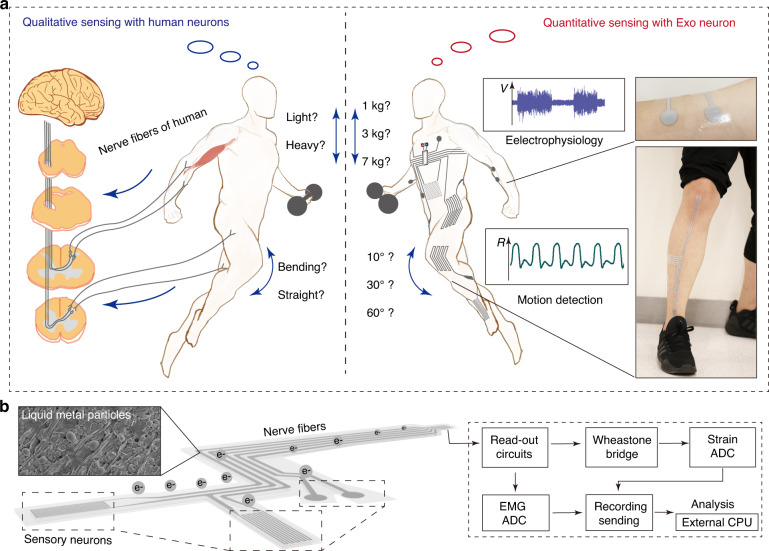
Illustration and structural design of the EEN device. **a** EEN for quantitative sensing compared with human neurons. **b** Schematic structure of the EEN

Proprioception facilitated by human somatosensory neurons is responsible for neuromuscular control and the activities of daily living. Through this kind of basic sensory modality, the perception and execution of movements are mediated by the central nervous system. In this way, changes in muscle length and tension, as well as joint motion and position, are captured by human somatosensory neurons. For example, when curling dumbbells or lifting items of different weights, humans can perceive whether they are heavy or not. During different exercises, humans can also distinguish their level of joint motion. However, this process is limited in an approximate and qualitative way (Fig. 1a, left). By attaching an EEN device to the skin of the human body, the quantitative sensing of the augmented somatosensory system can be realized for determining both joint motion and neuromuscular activities (Fig. 1a, right). The EEN applied on the skin serves to record electrophysiology and detect motion, and by analyzing the signals, specific parameters such as angles, angular velocity, and muscle loading can be obtained.

The basic structure of the EEN includes nerve fibers and sensory neurons, as illustrated in Fig. 1b. Among them, there are two kinds of sensory neurons (motion capture modules and electrophysiological electrodes). These sensory neurons with different functions have different patterns, including major motion capture modules for gait analysis and other modules, such as dual-channel electrodes, for electrophysiological signals. After being placed on a specific area of the skin (Fig. 1a, right), the EEN first senses the digitalized signals through the sensory neurons, and then the signals are passed onto read-out circuits along the nerve fibers. On the one hand, signals of joint motion are realized by the variation in resistance as a result of the deformation of skin and further processed by a Wheatstone bridge and strain ADC (analog–digital converter) and finally sent to an external micro CPU. The ADC is an analog–digital converter, which is usually combined with instrumental amplifiers to process signals. The original signal captured from the EMG and strain sensors are further processed and amplified by these circuit modules. On the other hand, an electromyogram (EMG) for analyzing muscle activity can be obtained by processing and analyzing the electrophysiological signals in the EMG ADC, as illustrated in Fig. 1b. There are two kinds of sensory neurons in the EEN: joint motion sensory neurons (Fig. S1a) and muscle activity sensory neurons (Fig. S2b).

**Fig. 2:**
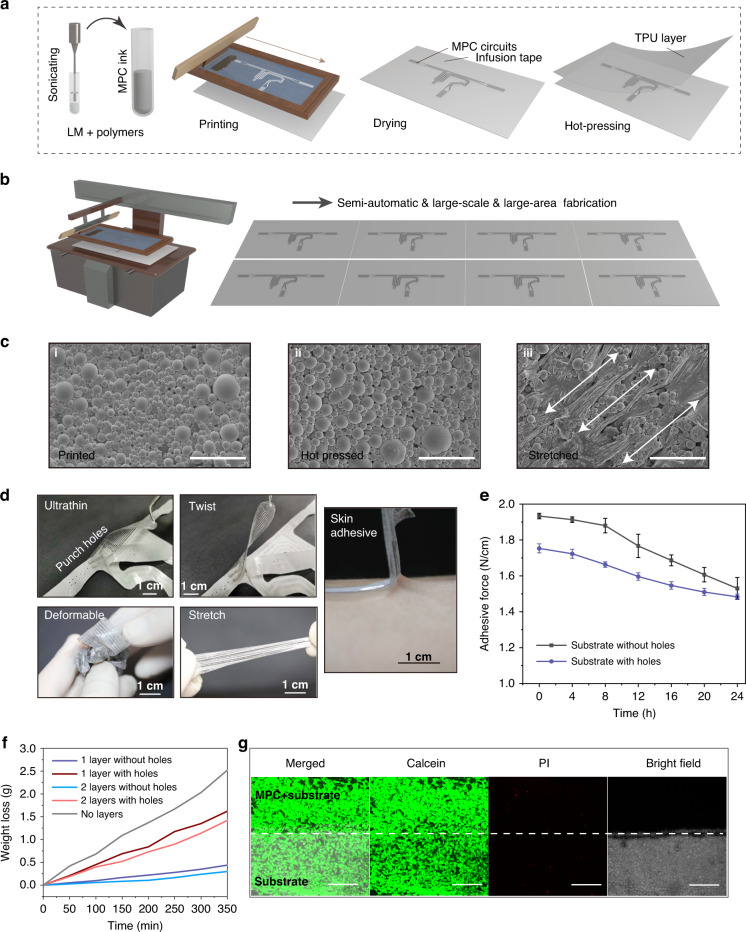
Fabrication and on-skin properties of the EEN. **a** Fabrication procedures (sonicating, printing, drying, and hot-pressing). **b** Illustration of large-scale fabrication. **c** SEM of liquid metal EGaIn microparticles: i, before hot-pressing, scale bar: 20 μm; ii, after hot-pressing, scale bar: 20 μm; iii, after stretching, scale bar: 10 μm. **d** Completed EEN with holes. It is ultrathin (<110 μm), stretchable and skin-adhesive (scale bar: 1 cm). **e** Adhesive force of the substrate with and without holes. **f** Perspiration test. **g** Cell viability test, scale bar: 200 μm

Figure 2a presents the simple and quick fabrication methods and on-skin properties of the EEN. First, MPC ink for circuit printing is obtained by sonicating the liquid metal (LM) GaIn alloy (4:1) and polymers in solution. The GaIn alloy (4:1) has properties that are similar to those of Galinstan and the eutectic gallium-indium alloy (a liquid state at room temperature with a viscosity of 2 × 10^−3^ kg/m/s, density of 6 g/cm^3^, and electrical conductivity of 3 × 10^6^ S/m). These materials are alternate choices for the fabrication of MPC ink instead of gallium with a melting point of 29.8 °C (solid at room temperature). Further steps of heating the gallium for sonication are avoided. A specific pattern for motion detection is printed in the infusion tape layer. A hot-pressing method was employed to add a protection layer at the end. By combining sectional hot-pressing and semiautomatic screen-printing methods, the limited size of spin-coating methods can be overcome, and large-scale EEN fabrication can be realized, as shown in Fig. 2b. The substrate layer is composed of a clinical infusion tape made of a commercial Hons Medical sticky film that is widely used for patients in hospitals for approximately several days. The clinical infusion tape as the substrate layer is thin (~50 μm), soft, stretchable, and comfortable when worn on human skin. The morphology of the MPC inside the EEN after hot-pressing basically remains the same as before (Fig. 2c). In addition, full stretching of the EEN in the axial direction is needed to tear the oxidation film of MPC microparticles (Fig. 2c iii), which facilitates the complete conductivity of the EEN. The addition of polymers in liquid metal ink is beneficial for anchoring the liquid metal particles to the substrate^25^. When stretching occurs, the stress of the substrate is transferred to the particles. This process breaks the oxide layer of particles and forms a conductive path between the particles. Figure 2d shows the basic properties of the EEN. It is soft, ultrathin, deformable, and stretchable and adheres to the skin. These properties meet the requirements for skin conformality^33^, which is essential for on-skin signal recordings. The ultrathin thickness (~110 μm) and sticky viscidity of the whole material ensure that it can conformally deform along with the skin, which gives it the ability to accurately sense the bending deformation and anti-interference of human joint motion, especially during strenuous exercise, compared to accelerometers. To accommodate perspiration under strenuous conditions and during long-term use, several holes with a radius of 0.8 mm are punched in the substrate (apart from the MPC part) of the EEN. The existence of holes in the substrate reduces the contact area, which further causes a slight decrease in the adhesive force for the skin, as shown in Fig. 2e. However, the substrate with holes still maintains a high adhesive force level in the long term for approximately 24 h due to the improved accommodation of perspiration (Fig. 2f). As shown in Fig. 2d–f, several holes are punched in the substrate, and a perspiration test is conducted. Normally, the aggregation of sweat affects the adhesive force. However, the holes in the substrate can release the majority of the sweat, as shown in Fig. 2f, and lower the rate of decrease in the adhesive force. The substrate still retains a high adhesive force above 1.5 N/cm to prevent detachment. Biocompatibility is also important for on-skin devices, especially for long-term use. Although the substrate is made of a clinically widely used infusion tape layer that can be used for at least 48 h without allergic skin reactions, the cell viability both in the substrate and liquid metal is further characterized. The calcein-stained live cells and bright-field (BF) images shown in Fig. 2g indicate that the cells grown in both the substrate and MPC parts have normal morphology. A few dead cells stained by propidium iodide can be seen in Fig. 2g. Thus, the EEN exhibits good biocompatibility.

The mechanism and effectiveness of the EEN for motion tracking is characterized in terms of three aspects: electromechanical performance, accuracy, and stability. Based on the principle that resistance changes in a fixed ratio with the deformation of soft electric circuits, the strain vs. ∆*R*/*R* should be linear for correct angular transformation. Normally, before the linear region of the ∆*R*/*R*-strain curve that can be used to measure the deformation, there is a small nonlinear region that should be avoided. To solve this problem, the strategy is to add a prestretching step to prevent the occurrence of the initial nonlinear region. Due to their high surface tension, gallium-based alloys have printing and patterning constraints^34^. Generally, gallium-based alloys are fabricated into micro/nanoparticles with an external nonconductive oxide layer^27^. After the printed circuit is fabricated, the oxide layer hinders the conductivity of the circuit. To activate conduction by stretching, the oxide film is partially torn, and conductive pathways are formed^25^. When it is stretched and used for motion detection in the initial stretch area of approximately 10%, the deformation has little effect on the increase in resistance, resulting in a nonlinear curve. The nonlinear curve shows an irregular relation that should be avoided. The motion capture module of the EEN is subjected to three different prestretching (0, 10%, 20%) treatments (Fig. S3a) before the test of stretching to 50%. It is obvious that there is a small nonlinear region at the beginning when the sensor is not prestretched, while the 10% and 20% prestretched sensors exhibit linear variation during the whole strain from 0 to 50%. Thus, this indicates that a prestretching of >10% is needed when attaching the EEN sensors to the skin for motion tracking.

Considering the complex scenario of sensors with various velocities, the ∆*R*/*R*-strain curves at different speeds are illustrated in Fig. S3b. To simulate the influence of the reciprocating deformation of the joint at different velocities on the sensor performance, the sensors are stretched to a strain of 50% at speeds of 5, 15, 30, 60, and 90 mm/s and then off-loaded. As the strain increases, the resistances of all sensors begin to enter the linear ascending region after a nonlinear rise of approximately 10%. In the two processes of strain increase and decrease, all curves basically coincide with themselves at 5, 15, 30, and 60 mm/s, and the slopes of the curves remain basically unchanged, except for a slight increase at 90 mm/s, which far exceeds the rate of deformation of human joints in daily exercise. Considering their application on limbs, the sensors should be able to bear a large deformation. Compared with liquid metal-based strain sensors, carbon nanomaterial-based strain sensors generally have lower stretchability (<200%) and conductivity (0.1–100 S/m)^8,35^. Liquid metal-based strain sensors have high stretchability (>500%) and excellent repeatability and conductivity.

**Fig. 3:**
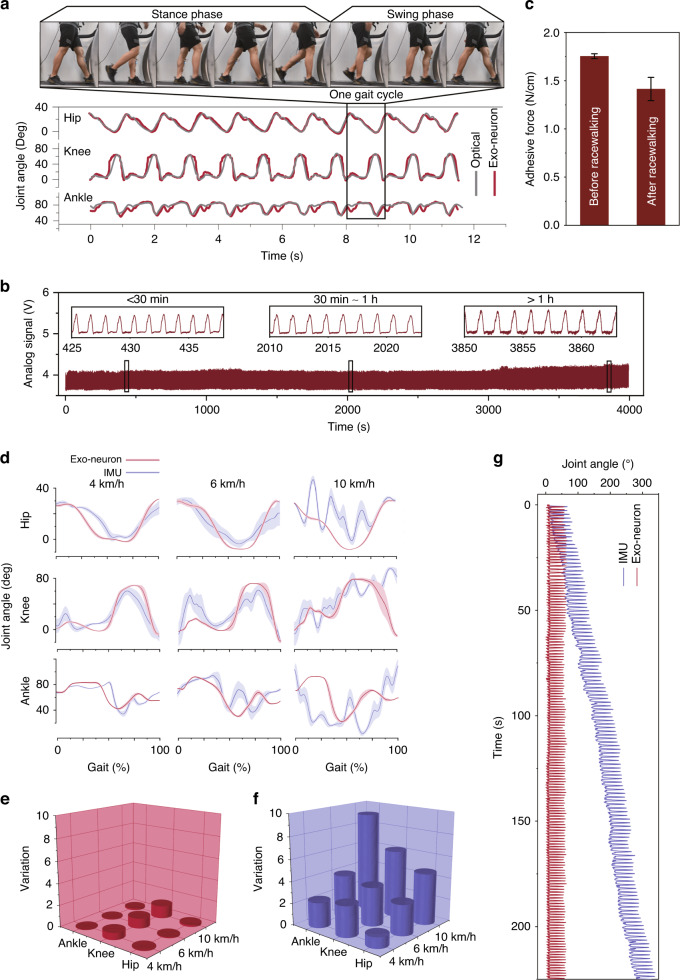
Characterization of the motion capture module of the EEN for motion monitoring. **a** Gait cycle of three joints when walking at 4 km/h captured by the motion capture module of the electronic exoneuron and optical motion capture system. **b** Real-time monitoring of the racewalking of the athlete by the EEN applied on the skin. **c** Adhesive force before and after exercise. **d** Stability comparison of EEN and IMU methods for capturing the motion of lower limbs from slow walking to running. **e** Coefficient of variation of three joints during the motion of lower limbs captured by the EEN method. **f** Coefficient of variation of three joints during the motion of lower limbs captured by the IMU method. **g** Drift effect comparison of EEN and IMU methods

To demonstrate the function of tracking body motion by the EEN, soft motion capture modules of the EEN are placed at three different joints in the lower extremities: the hip, knee and ankle. In a single gait cycle, the signal changes of various joints are different and have their own waveforms with specific shapes. In this case, the EEN shows potential for use in the gait analysis of human lower limbs for rehabilitation and sports analysis. This can be identified in real-time from the extracted frame of a single cycle, as shown in Fig. 3a.

Figure 3a reveals the results of tracking lower limbs when the participant is walking on a treadmill at a velocity of 4 km/h, and the data of several cycles are presented. The red line represents the changes in the angles of three joints on the right leg measured by EEN during walking and indicates that the bending of different joints and gait cycles during movement can be directly (raw data) and stably (without the integration of error) recognized by the EEN without further filtering processing, such as with Kalman or Particle Filters.

We can see that the hip joint angle waveform is mainly U-shaped with the characteristics of a single wave trough, while the knee joint angle variation occurs in two distinguishable peaks, first a small one followed by a larger one, which is caused by the bending of the knees two times during one single cycle of walking. In addition, the waveform of the ankle joint angle first slowly appears with a high peak followed by a lower peak. Moreover, a comparison is made with the gold standard method (gray line) optical motion capture system as an accurate standard. Generally, the angles of the three joints captured by the EEN fit the standard line (Fig. 3a), and the relation between the angles and signal sensors is shown in Supplementary Section 1, Fig. S1 and Fig. S4. Since this system is sensitive and directly affected by the deformation of the skin, we infer that the errors and the extra unaccounted troughs that the optical motion capture system cannot detect are caused by the complex tiny changes of skin that the optical camera cannot sense. However, we can obviously distinguish the gait cycle of three joints, especially the hip and knee joints, without further filtering and modeling. Otherwise, this system is a possible tool that provides sufficient movement information to analyze the various gait states of the participants, such as walking, running, and jumping.

In tracking body motion exercises, the motion capture module of the EEN also exhibits practical repeatability. To prevent the accumulation of sweat, several holes (0.8 mm radius) are punched in the EEN to accommodate perspiration. Considering the side effects of these holes in the EEN on repeatability and function, further characterization is carried out. First, the EEN is attached to a silicone film with an elastic modulus similar to that of human skin and stretched to a strain of 50% more than 500 times for 2500 s on an electric rail with basically no change in resistance (Fig. S5a). For more complicated situations encountered in actual utilization and considering that the knee joint can be regarded as a representative joint of the largest deformation movement in the three joints of the lower limbs, the EEN is further attached to the surface of the knee joint for practical testing. The ∆*R*/*R* of the EEN is recorded every 2 h at two extreme cases of deformation (high knee lift and standing) in half a day of daily activities. After 12 h, the nuanced response of the resistance is observed, as shown in Fig. S5b, and the resistance decreases slightly, which is due to the repeated stretching movement of the joints. The liquid metal particles of the MPC are more densely connected to form a wire path, and the direction of the path becomes more consistent. Specifically, for practical detection for racewalking athletes for at least 1 h (Fig. 3b), the changes in the knee joint recorded in real-time also indicate good stability and repeatability. A small variation (<4.1%) occurs above 1 h, which may be a result of the muscle tremor of the athlete due to long-term walking. Thus, the EEN shows potential use in sports for a long period (at least 1 h). Adhesive force is further characterized before and after long-term exercise. The results shown in Fig. 3c demonstrate that the EEN still maintains a high adhesive force on the skin. In addition, there is no characteristic difference in the knee joint angle data of the left and right legs measured by the EEN (Fig. S3c).

As an alternative approach to optical motion capture for analyzing human motion, the IMU method can overcome the limitations of fixed space sites, but the EEN method has better stability than the IMU method without the need for complicated filtering. The on-skin stability of the EEN compared with that of the IMU is further characterized by comparing the coefficient of variation with the deviation of the IMU. The coefficient of variation describes the extent of variability in relation to the mean. It is used to measure the dispersion of data distributions^36^. Here, it is employed to evaluate the variation and stability between the IMU and EEN. In addition, in the actual gait analysis experiment, we measure the changes in three joint angles (hip, knee, and ankle) under different motion kinetic conditions, including slow walking, fast walking, and strenuous running. First, we segment the period of each joint under specific motion conditions and then superimpose them into one cycle. As shown in Fig. 3d, it can be clearly and intuitively seen that the signal recorded by the IMU, represented by the blue line, shows much more intense fluctuations than the data collected by the EEN, represented by the red line. Especially when the activity becomes increasingly intense (from slow walking at 4 km/h to fast running at 10 km/h), the data collected by the EEN still maintain a relatively stable and clear waveform, while the data collected by the IMU fluctuate more and more intensely, and the drift becomes increasingly obvious. The fluctuation is illustrated in Fig. 3g. To verify the stability in the long term, Fig. 3g shows that the raw data from the IMU (blue line) without filtering and calibration exhibit data shifting (up to 300° within 200 s), while the raw data captured by the EEN (red line) show excellent stability (no shifting). The specific time points with the largest fluctuations in each superimposed cycle under specific motion conditions are selected, and then the standard deviation of the measured signal at that time point is calculated and divided by the mean, and then the coefficient of variation is finally obtained, as shown in Fig. 3e (EEN) and Fig. 3f (IMU). From the red cylinders, we can see that the coefficient of variation of the EEN is basically less than 1. However, the coefficient of variation of the IMU represented by the blue cylinders is much larger than that of the EEN. Especially for the knee and ankle joints, the coefficient of variation of the data measured by the IMU is almost 6–8 times larger than that measured by the EEN. Since two IMU sensors need to be placed at two ends of the joint when measuring the angle of a single joint while the EEN is conformally attached to the skin outside the joint, IMUs undergo greater fluctuations from the leg muscles during exercise, resulting in the raw data measured by IMUs being more susceptible to fluctuations during exercise, especially strenuous exercise. For daily common exercises, such as rope skipping and squat jumping, as illustrated in Fig. S6a, b, the red line is the signal from the knee joint measured by the EEN, and every jump can be clearly distinguished with almost no signal blurring from noise. However, the data from the IMU represented by the blue line is disorganized, and each jump cannot be distinguished.

**Fig. 4:**
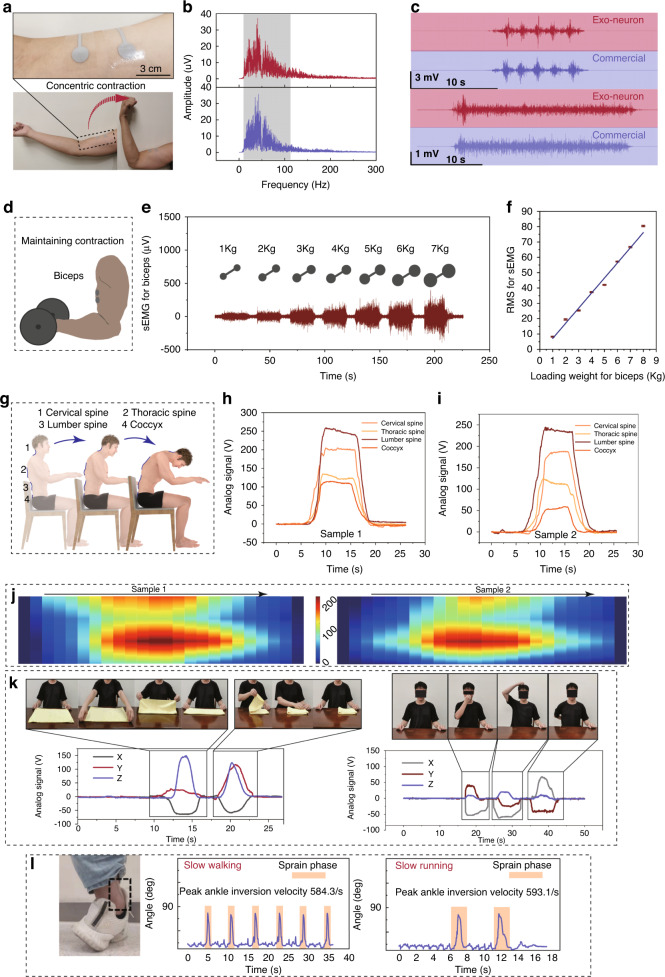
Quantitative sensing by the EEN for rehabilitation. **a** Electrodes of the EEN during the concentric contraction of the biceps. **b** Frequency spectra of the EMG signals of the EEN and commercial electrodes. **c** Comparison of the EMG signals during dynamic contraction (upper) and isometric contraction (down) of the EEN and commercial electrodes. **d** Illustration of maintaining the contraction of the biceps with dumbbells. **e** sEMG signals of the biceps during tasks with different muscle loadings (1–7 kg). **f** RMS of the sEMG signals collected under different weight loadings. **g** EEN positions for spine monitoring. **h**, **i** Recording signals of spine bending for two samples. **j** Heatmap of spine bending monitoring. **k** Signals of the shoulder joint under different tasks for rehabilitation evaluation. **l** Quantified evaluation for the sprain of the ankle joint

Quantifying muscle activity and joint ability is necessary for proprioception rehabilitation. Failure in proprioception may have a profound effect on muscular control and daily activities. The human somatosensory system cannot be used to quantify motion ability, especially when evaluating whether injured people have the ability to return to normal life. Rehabilitation trainers need quantifiable auxiliary assessment tools to judge the abilities of patients to realize evidence-based recovery guidance. The EEN shows great potential in this regard by quantifying both muscle activity and joint motion. The performance of electrodes in the EEN for EMG collection is further characterized, as shown in Fig. 4a–c. A pair of EEN electrodes are affixed parallel to the outer skin of the biceps of the right arm. The electrical signal collected during the continuous contraction of the biceps brachii muscle is processed, and the distribution of the amplitude with the frequency change can be clearly seen in Fig. 4b, which indicates that the collected signal is concentrated at approximately 40 Hz, confirming the effectiveness of the EEN electrodes for collecting EMG signals. The frequency range of an EMG signal^37^ is generally between 0 and 500 Hz, and the dominant range of our results is approximately 20–120 Hz. This characteristic can be used to distinguish the EMG signals. A participant performs concentric contractions and bends their right arm (Fig. 4a), including 5 consecutive dynamic contractions (Fig. 4c, top) and static continuous contractions (Fig. 4c, bottom). The data is recorded and compared with data from commercial electrodes. The signal-to-noise ratio shows competitive performance compared to that of the commercial electrodes (Fig. 4c).

Biceps weight training, in which subjects perform dumbbell bending tasks with gradient weights of 1–7 kg while maintaining static contraction (Fig. 4d), is selected as a quantitative functional test task. The sEMG signals of the biceps with different weight loadings are recorded, as shown in Fig. 4e, which shows an obvious increase. Figure 4f shows that the calculated RMS of the EMG signals presented a linear variation, which suggests that this method is a potential evaluation method for quantification. For people who often sit in front of a computer, improper sitting posture and long-term spine bending lead to spinal-related neurological diseases, which affects people’s daily life over time. The EEN is placed on four parts of the spine: the cervical spine, thoracic spine, lumbar spine, and coccyx. When subjects bow their heads and bend their backs, characteristic signal responses occur, as shown in Fig. 4h, i. Overall, the lumbar spine shows the largest flexion during the whole process, as shown in the heatmap (Fig. 4j). In this way, the level of bending of the spine can be quantified, and the EEN can be further regarded as a preventative tool for correcting sitting postures. The possibility of evaluating the ability of the shoulder joint in the rehabilitation of upper limbs is further tested (Fig. 4k). Folding towels and touching three points (nose, head, and back) are considered common tasks in rehabilitation evaluation. These simple movements cover the basic needs of normal people in daily life. Signals from the right shoulder (three directions X, Y, Z) collected by the EEN present a clear variation during these tasks. Combining these observations and quantified data, a more comprehensive method for the evaluation of patients under rehabilitation can be realized. Furthermore, ankle sprains are highly prevalent both in sports and daily life and have a high risk of recurrence. The lateral ankle sprain is the most common form of ankle sprain. The hazardous speed of peak ankle inversion is approximately 481–1752 deg/s. The EEN can be used to monitor the peak inversion and quantify the velocity. When the participant performs several trials of simulated sprain motions during slow walking and slow running, the signals of peak inversion are recorded (Fig. 4l). The sprain phase is obvious during the exercises. In the future, we believe this monitoring method can be integrated with electrical stimulation to the lateral shank muscle to further prevent injury.

**Fig. 5:**
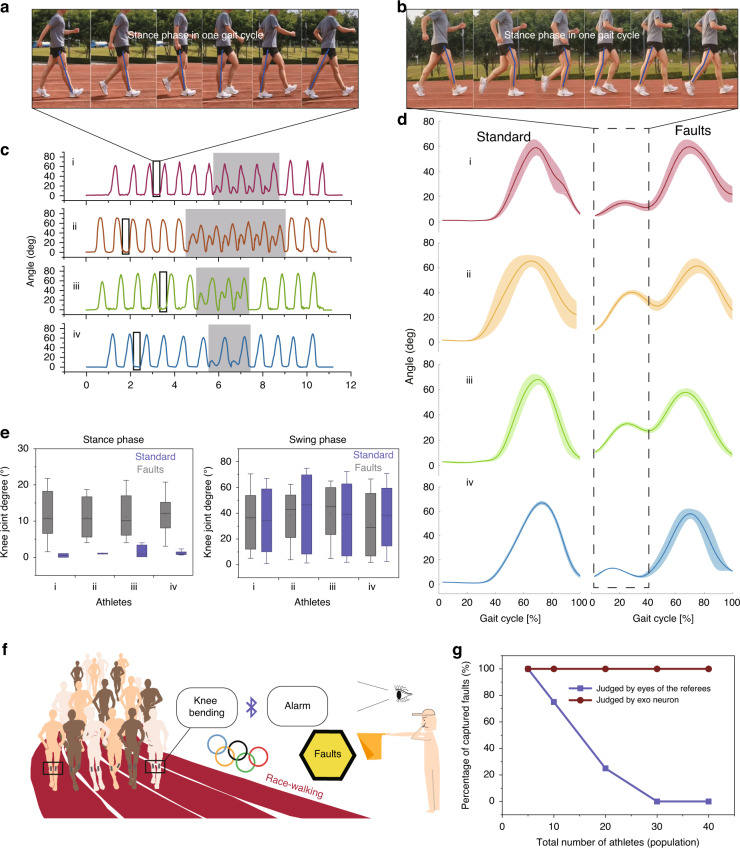
Objective detection in racewalking by the EEN. **a** Illustration of standard racewalking of the right leg in the stance phase during one gait cycle. **b** Illustration of a foul in racewalking of the right leg in the stance phase during one gait cycle. **c** Knee joint angles of four expert athletes during racewalking with several random fouls. **d** Results of gait analysis of four expert athletes. **e** Statistical comparison in the stance phase and swing phase. **f** Illustration of racewalking with EEN. **g** Comparison of captured faults by referees and EEN

As a multiparticipant competitive event of the Olympic games, racewalking requires many referees to use their eyes to guarantee fairness. According to the rules of racewalking, athletes cannot bend their knees during the stance phase (the moment from when the heel of the leg touches the ground to when the tip of the toe leaves the ground) of the gait cycle (shown in Fig. 5a). This rule is used as the criterion for distinguishing between running and walking. In competitive racewalking, such as that in the Olympic Games, faults are generally determined by the observation of judges. The motions of thousands of athletes are typically blocked by each other and are often disputed. Motion capture by video is not possible, as this would require a huge system of cameras to cover the entire space (between 20 and 50 km); motion capture by IMUs is unpractical, as they add too much weight to the athletes. Thus, an objective tool that can both be imperceptible to athletes and assist in judging athletes’ faults is urgently needed.

In this experiment, the EEN is attached to the right knee of four Olympic racewalking athletes (Table 1), and the possibility of using EEN to assist in monitoring racewalking faults and related training corrections is explored. Four different participants are required to act faults randomly during normal racewalking. The EEN is used to monitor and record the changes in the angle of the participants’ right knee.

**Table 1:** Information of the four expert racewalking athletes

Participants	Height [m]	Gender	Training experience [year]
i	1.80	Male	8
ii	1.58	Male	3
iii	1.56	Female	2.5
iv	1.65	Female	4

Several gait cycles (Fig. 5c) that included faulty actions are selected. The results show that despite the different racewalking subjects, the gait cycles of their knee joints all show a specific pattern: first, they maintain an almost horizontal state in the stance phase, and then a peak appears in the swing phase. When the faulty action (Fig. 5b) occurs, the gait cycle of the knee joint changes obviously (as shown by the shadows in Fig. 5c) with a bimodal state in each cycle. Multiple cycles are superimposed to obtain a superimposed diagram (Fig. 5d) of two states. In the standard racewalking state (Fig. 5a), we can see that the knee angle remains almost unchanged at approximately 0° in the cycle diagram. At this time, the test leg remains in the state of knee extension, then it enters the swing phase in the following period of time, and the knee flexion reaches a peak of approximately 70°. In the foul state, we can see that the knee joint bends first, and the signal rises to a smaller peak; later, the knee joint bends again to elicit a larger peak in the swing phase (Fig. 5d). We further analyze two phases of one gait cycle for all athletes, as shown in Fig. 5e. As athletes are different people with certain personal gait cycles, there are differences among all athletes. However, the data from all athletes show the same characterization in both the standard state and fault state. In the stance phase, the gray box line presents a range of angles that is 10 times larger than that of the blue box line, which indicates that there is a drastic fluctuation when faults occur. The swing phase shows no large distinction between the standard and fault states. Thus, the stance phase can be regarded as a phase for reliable and effective judgment.

The potential judging ability in daily training with different numbers of athletes is further tested (Fig. 5g). When the total number of athletes increases, it becomes increasingly difficult for one referee to detect all faults (the percentage of detectable faults is approximately 80% for 20 athletes). In contrast, the EEN can detect 100% of the faults of 20 athletes during racewalking. In this way, combined with Bluetooth data transmission or wearable alarms, it can be used as an objective auxiliary judgment method to assist referees in racewalking events. Thus, it facilitates a wide range of possibilities for using objective tools in the daily training of racewalking and Olympic competitions (as shown in Fig. 5f).

## Conclusion

In this work, we developed an electronic exoneuron for quantitatively sensing the augmented somatosensory system. It accurately sensed both physical and electrophysiological signals and detected faults during racewalking by Olympic athletes. It overcame the constraints of the physical space and high cost of optical motion capture systems and further avoided the data shifting and complicated postprocessing required by wearable IMU sensors based on accelerometers. Avoiding complex synthesis and fabrication methods, we combined hot-pressing and screen-printing strategies to fabricate the EEN in a straightforward way at a low cost of approximately 0.8 dollars per device. Directly attached to the skin outside the joints, the EEN with conformal properties, great repeatability and stretchability, and a thickness of only approximately 110 μm quantified muscle activity and joint motion during daily activities, even under strenuous exercises for at least 1 h. Throughout the skin of the human body, the embedded MPC served as electric nerves for sensing and signal transmission, which showed great potential in the long-term monitoring of human activity in health and sickness. Thus, this device is a potential tool not only for sports competition but also for medical rehabilitation and health care monitoring and as a soft interface for soft robots and the virtual reality/augmented reality of the Metaverse.

## Materials and methods

### Liquid metal–polymer conductor (MPC) ink preparation

First, a PVP solution was made by adding 1 g PVP (polyvinyl pyrrolidone, Aladdin, Mn = 1,300,000, China) into 19 g hexyl alcohol (98%, Macklin, China) and stirring for 24 h. Then, the liquid MPC ink was made by sonicating 3 g liquid metal (gallium–indium alloy, Ga: In = 4:1, melting point 15 °C, Hawk, HK3284, China) in l mL PVP solution for 60 s (5 s on, 5 s off) at an amplitude of 18%. A probe sonicator (S450D, Branson, USA) was employed during sonication.

### Substrate preparation

The infusion tape layer was composed of a medical PU infusion tape layer (HS096, Hons Medical, China) clinically used for long-term patient care. The infusion taper layer was cut and customized into a designed shape with a specific length and width as the substrate with a sticky side. The protective layer was a hot melt TPU film (DS3412, Tunsing, China) with a thickness of approximately 0.05 mm and a melting point of approximately 125 °C.

### Fabrication of the EEN

The motion capture module sensor was fabricated by first using the screen printing method to print the liquid MPC on the infusion tape layer in a linear network shape and then drying it in an oven at 80 °C (DHG-9420A, Yiheng Scientific Instrument Co., Ltd., China) for 5 min. Once dried, the electric connection points of the motion capture module were attached by a polyimide conductive contact film based on a multilayered method, and then a hot melt TPU film was hot-pressed (G311, FREAMC, China) at 125 °C (with pressure ~ 250 kPa) for 15 s (to maintain a good quality of the substrate, as shown in Fig. S9). The release film on the other side of the TPU film was peeled off after it cooled down. To reserve a port for an electric connection, the hot melt TPU film at the connection point was peeled off. Additionally, the liquid metal electrodes were screen-printed as circles with a radius of 6 mm on a polyethylene terephthalate (PET) film. After drying in the oven at 80 °C for 5 min, the sticky side of the transparent film dressing was applied and hot-pressed at 125 °C (with pressure ~250 kPa) for 15 s (G311, FREAMC, China). Thus, the liquid metal-polymer conductor was transferred to the sticky side of the infusion tape layer, and in this way, the electrodes of the EEN were fabricated. Third, neuro-like connection soft circuits throughout the surface of the human body at a large scale were fabricated by printing 6 mm width circuits on the infusion tape roll as large as the required connection length. The thickness and stress‒strain curve of the whole EEN layer are shown in Figs. S7–S8. The circuit design is shown in Fig. S10. The electronic components used in this work included instrumental amplifiers (AD627), LM324DR, and MF4054 for signal processing. MicroQTJ and N76E003AT20 were employed for wireless communication.

### Posted on skin preparation

Once the fabrication of the EEN was completed, a fully axial tensile strain (approximately 50%) was needed to tear the oxidation shell of the liquid-metal microparticles of MPC to form conductive circuits among the particles (Fig. 2c) and further induce the conductivity of the whole EEN. Several holes were punched (with a 0.8 mm radius Holer Puncher, OEM-0.8, Mayer, China) into the EEN away from the position of the circuits. Especially for the motion capture module, 10% prestretching at the joint deformation axis direction was applied before it was applied to the epidermis outside the joint angle, as discussed before and illustrated in Fig. S3a.

### Characterization of EEN

The morphology of EEN was characterized by the Scanning Electron Microscope (Zeiss, Merlin) at SUSTech Core Research Facilities. Fluorescence images were taken by Nikon Confocal A1R with FLIM (Nikon, A1R + Symp64) at SUSTech Core Research Facilities. For the motion capture module, we used a linear guide slide (FSL_40, FUYU, China) to apply tensile strains on samples and used an electrochemical station (1040C, CHI, China) (the amperometric *i*–*t* curve was recorded at a potential of 0.05 V) to record the electrical signals of the samples under deformation. We applied 50% unidirectional tensile strain on the samples for 500 cycles. The optical motion capture system (Motion Analysis, Raptor-4S, USA) was employed as the gold standard for gait analysis, and IMUs (6050, Taike) were mounted on the lower limbs in the sagittal plane. Regarding the electrodes, we compared them with Ag/AgCl commercial electrodes of the same size and recorded the signal-noise ratio, and the electrodes were connected to a g.HIamp multichannel amplifier (G.Tek, Austria) to obtain a bipolar sEMG signal with a sampling frequency of approximately 1200 Hz through an analog notch filter at 48–52 Hz.

### Medical ethics

All human experiments were conducted with approval from the Medical Ethics Committee of Southern University of Science and Technology (approval No. 2021JCY055).

### Supplementary information

Supporting information

## Data Availability

The data are available from the corresponding authors upon reasonable request.
