# Supplementary material for: Electronic exoneuron based on liquid metal for the quantitative sensing of the augmented somatosensory system
Source: Microsyst Nanoeng. 2023 Sep 15;9:112. doi: 10.1038/s41378-023-00535-x (PMC10504372; doi:10.1038/s41378-023-00535-x)
Supplement: Supplementary file 1 — Supporting information [file 41378_2023_535_MOESM1_ESM.docx]

Supporting Information

**Electronic Exoneuron Based on Liquid Metal for the Quantitative Sensing of the Augmented Somatosensory System**

*Jin Shang, Lixue Tang, Kaiqi Guo, Shuaijian Yang, Jinhao Cheng, Jiabin Dou, Rong Yang*, Mingming Zhang*, and Xingyu Jiang**

* Corresponding author, E-mail: [jiang@sustech.edu.cn](mailto:jiang@sustech.edu.cn); [zhangmm@sustech.edu.cn](mailto:zhangmm@sustech.edu.cn); yangr@nanoctr.cn

The PDF file includes:

Supplementary Section 1: Stimulation and calculation of relation between angles and the sensor signals measured by EEN.

Fig. S1. Illustration of the deformation in the knee joint.

Fig. S2. Structure of the sensory neurons of EEN.

Fig. S3. The electro-mechanical performance of EEN.

Fig. S4. Illustration of the relation between angles and measured signals.

Fig. S5. Repeatiblity of the EEN.

Fig. S6. Comparison between the EEN and IMU methods for capturing rope skipping and squat jumping.

Fig. S7. Thickness of the EEN layer.

Fig. S8. Stress-strain curve of the EEN.

Fig. S9. Optical morphology of EEN after hotpressing.

Table S1. Information of the four expert racewalking athletes.

Fig. S10. Circuit design of the detection channel for racewalking and lower limb detection.

**Section 1. Stimulation and calculation of the relation between angles and the sensor signals measured by the EEN.**

Take the knee joint as the exmple for illustration, we simplified the deformation of EEN on knee joint shown in fig S1. The metal polymer conductors embedded in sensor of Exo-neuron is patterned as linear lines and attatched on the knee joint. We assumed that the sensor is like a linear resistance ***R*** and the initial length of it is ***L0*** and the angle of bending is $\boldsymbol{\alpha}$. The process of flexion and extension can be seen as a change in arc length with radius ***r*** which is defined under the specfic joint size of certain person. The final length of the sensor is defined as ***L***. As we discussed in Fig.3B, the ***∆R/R***-Strain($\varepsilon$) curve is regarded as a linear line after a 10% pre-stretching. So, basically we can get the function and $m$ is a constant coefficient

|  | $\frac{\Delta R}{R}=m\varepsilon=m(\frac{L-L0}{L0})$ | (1) |
| --- | --- | --- |

The length of the sensor is described as the function with the arc length

|  | $L= \alpha r$ | (2) |
| --- | --- | --- |

Combining function (1) (2), we can get the simplified relation between resistance and angles

|  | $\alpha=\frac{L0}{mr}\frac{\Delta R}{R}+\frac{L0}{r}$ | (3) |
| --- | --- | --- |

According to the formula (3), we can see that the $\frac{L0}{mr}$ and $\frac{L0}{r}$ are constants.


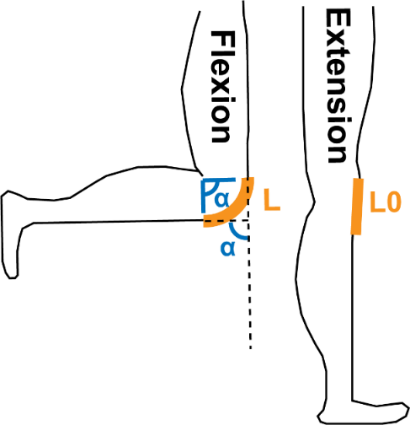


**Fig. S1 Illustration of the deformation in the knee joint.** Exo-neuron is attatched on the knee joint and the left shows the variation of angles during flexion state and the right shows the extension state.


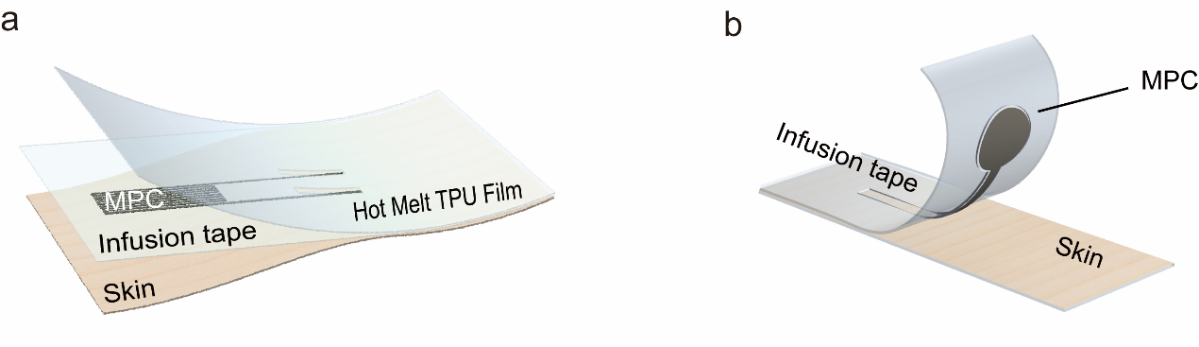


**Fig. S2 Structure of the sensory neurons of EEN. (a)** Joint motion sensory neuron. **(b)** muscle activity sensory neuron.


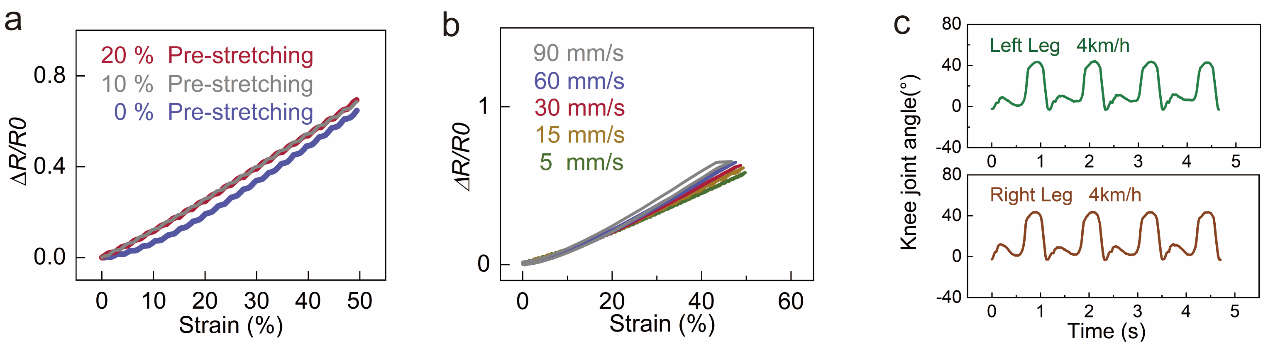


**Fig. S3 Electromechanical performance of the EEN. (a)** *∆R/R* of motion capture module of the EEN versus tensile strains from 0 to 50 % with different pre-stretching. **(b)** *∆R/R* of motion capture module of the EEN versus tensile strains from 0 to 50 % at various velocities. **(c)** Monitoring for knee joint angles of different legs when walking.


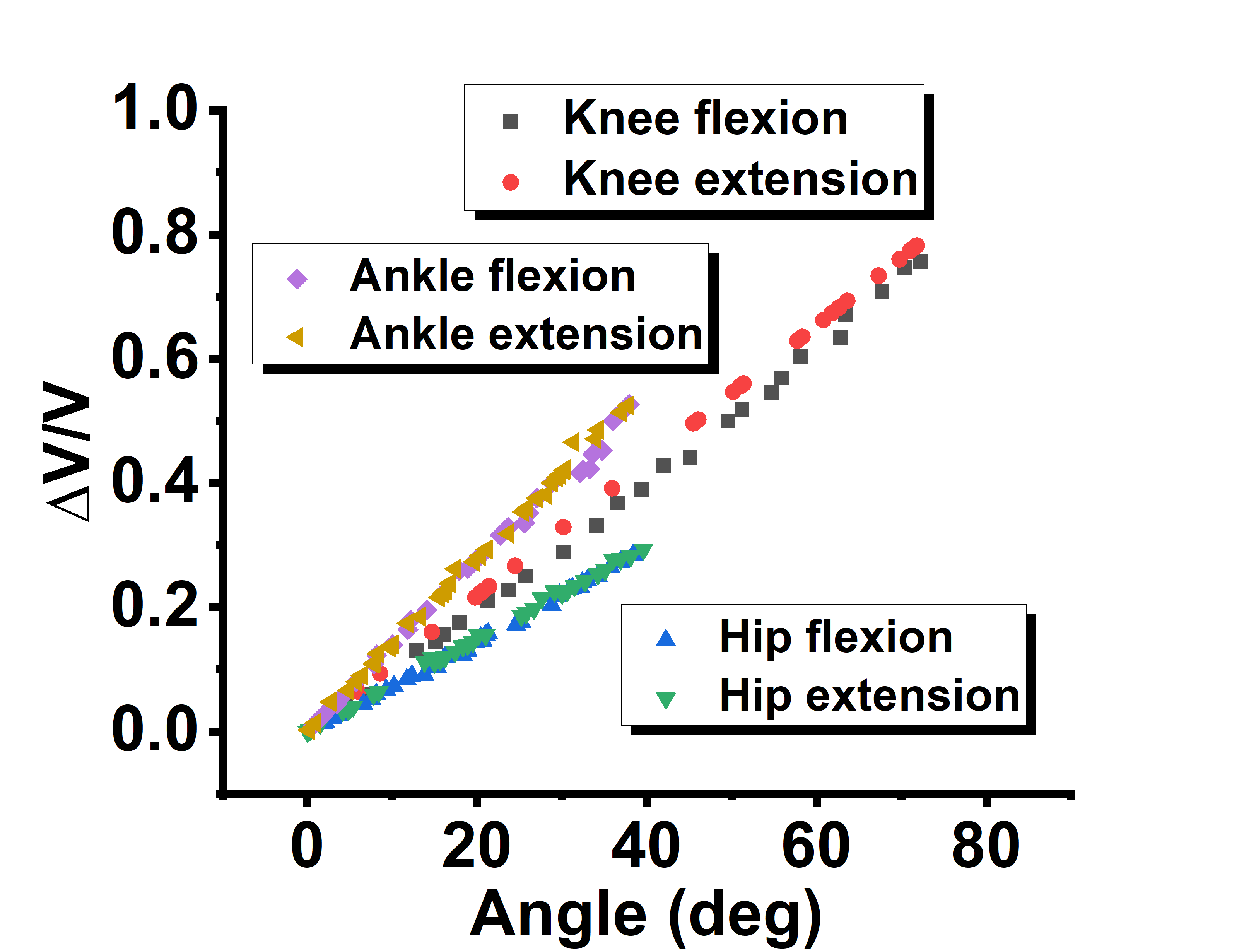


**Fig. S4 The illustration of the relation between angles and measured signals.**


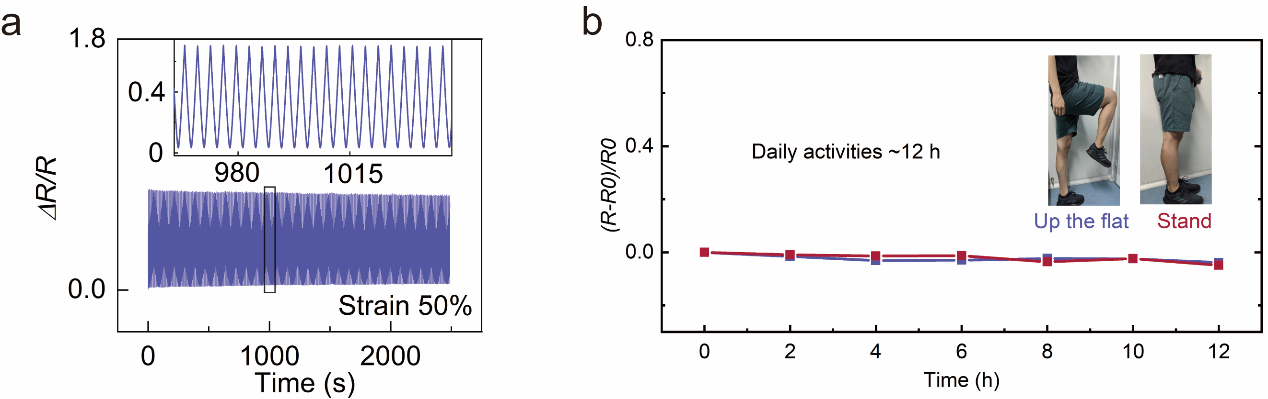


**Fig. S5 Repeatiblity of the EEN. (a)**Real-time monitoring of motion capture module of the EEN by stretching from a strain of 0 to 50 % around 500 cycles. **(b)** Variation of motion capture module of the EEN versus time from 0 to 12 h in one day.


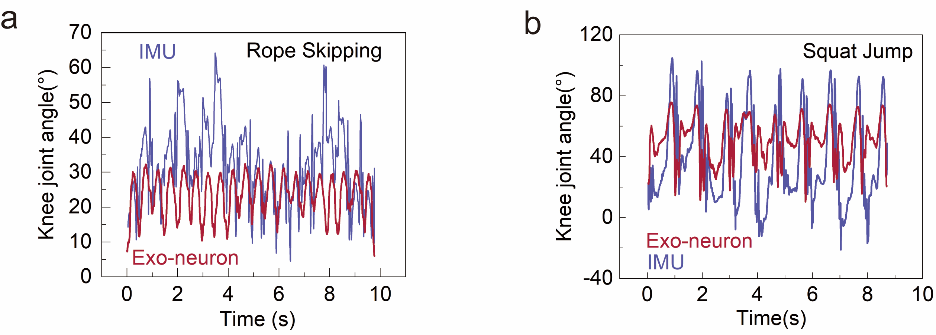


**Fig. S6 Comparison between the EEN and IMU methods for capturing rope skipping and squat jumping. (a)** Rope skipping. **(b)** Squat jump.


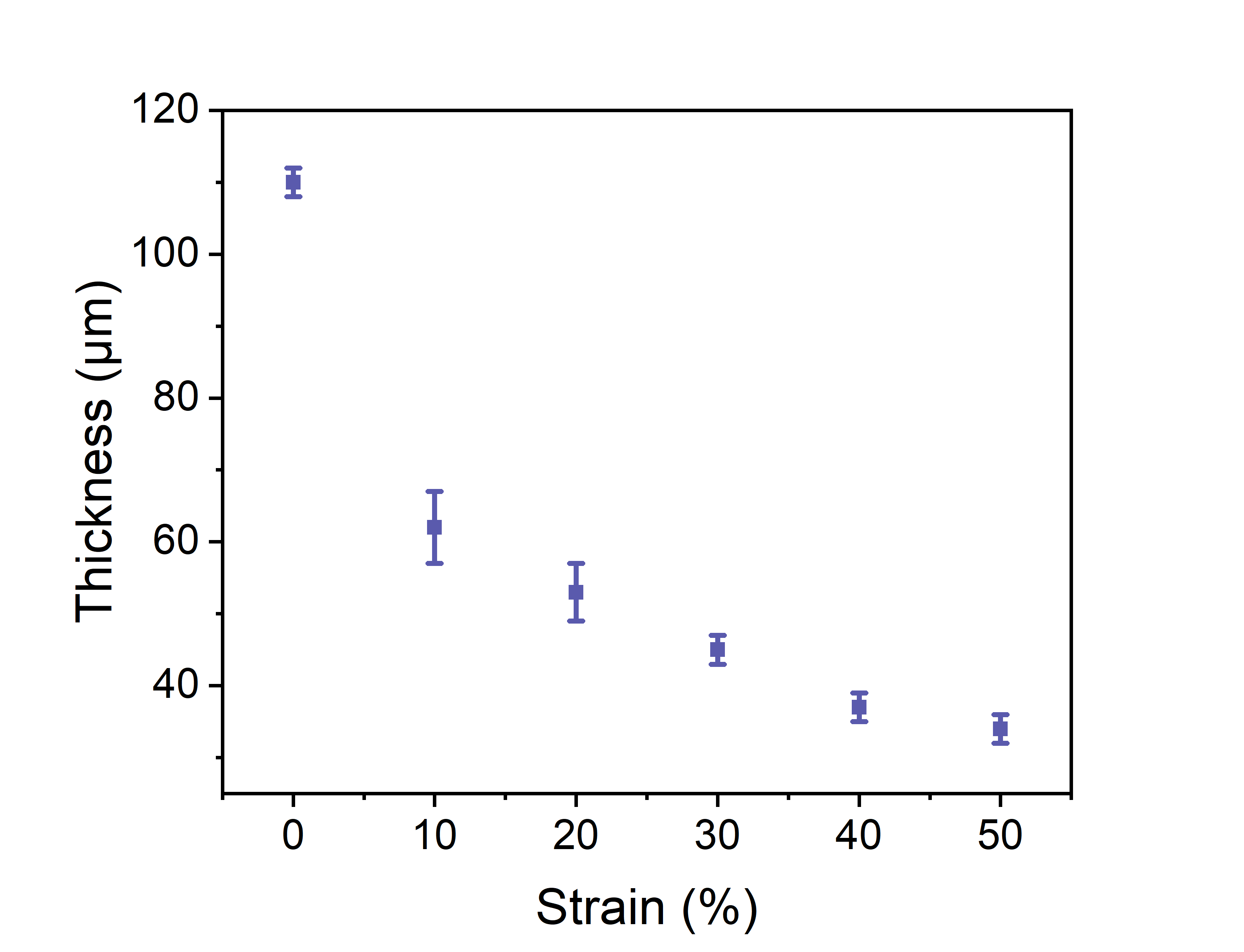


**Fig. S7** **Thickness of the EEN layer.**


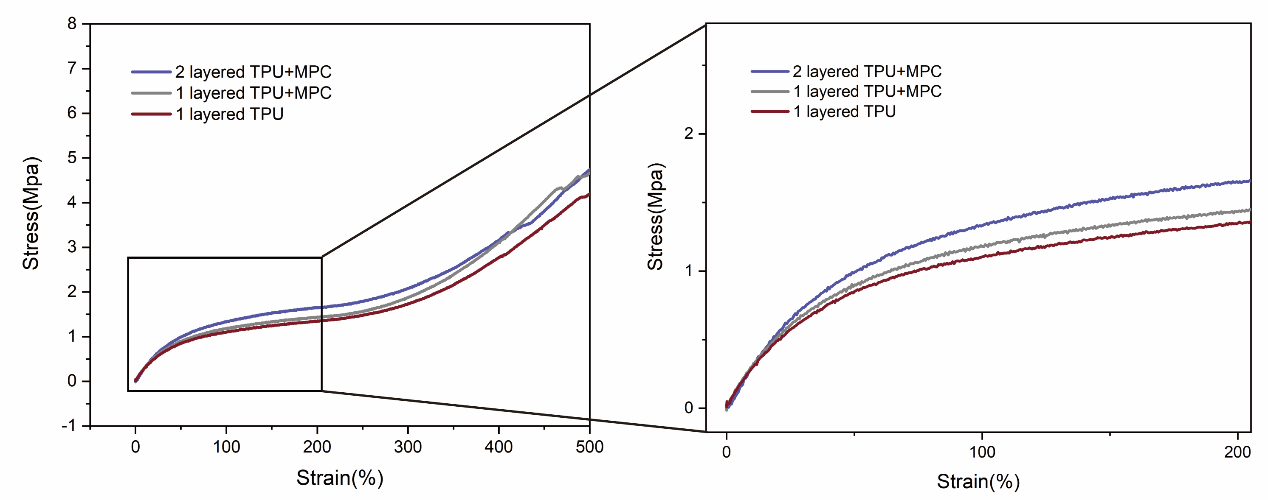


**Fig. S8 Stress-strain curve of the EEN.**


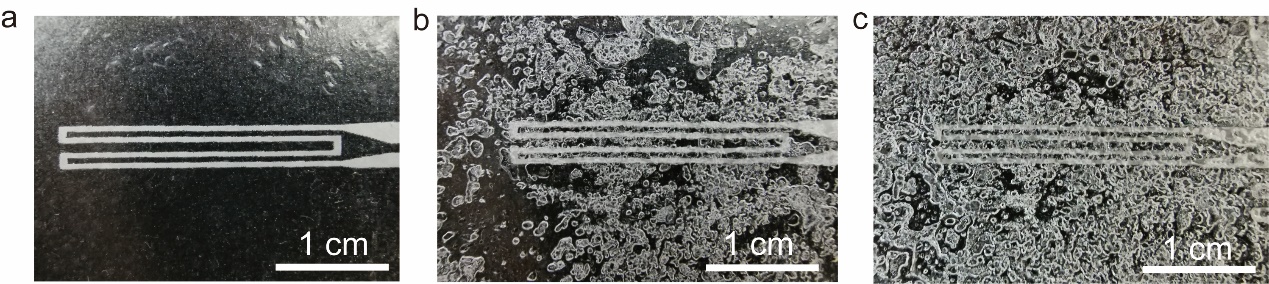


**Fig. S9 Optical morphology of EEN after hotpressing. (a)** Hot- pressed at 125℃ within 15 s. **(b)** Hot- pressed at 160℃ within 15 s . **(c)** Hot-pressed at 160℃ above 15 s.

Table. S1 Information of the four expert racewalking athletes

| **Participants** | **Height [m]** | **Gender** | **Training experience [year]** |
| --- | --- | --- | --- |
| *i* | *1.80* | *male* | *8* |
| *ii* | *1.58* | *male* | *3* |
| *iii* | *1.56* | *female* | *2.5* |
| *iv* | *1.65* | *female* | *4* |


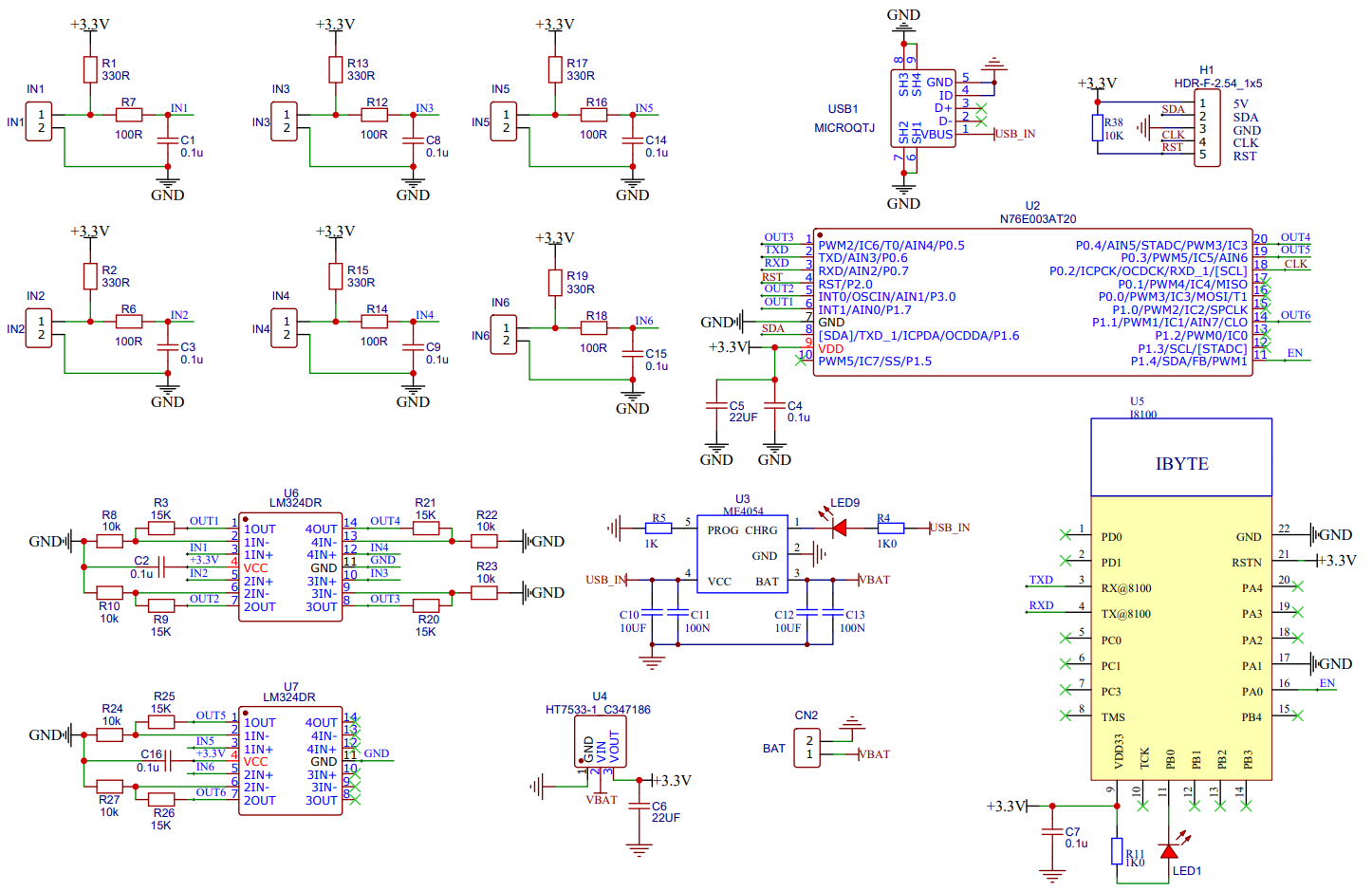


**Fig. S10 Circuit design of the detection channel for racewalking and lower limb detection.**
